# Does LLM translation align with translation universals? A cross-genre simplification study on English-Chinese translation based on dependency grammar

**DOI:** 10.1371/journal.pone.0324830

**Published:** 2025-06-02

**Authors:** Zeyuan Jiang

**Affiliations:** School of Foreign Languages, Qingdao University, Qingdao, Shandong, China; University of Kurdistan Hewler, IRAQ

## Abstract

This study examines the presence of simplification, a translation universal (TU), in English-to-Chinese translation by comparing the Mean Dependency Distance (MDD) and Mean Hierarchical Distance (MHD) of Crowdsourcing human translations, Large Language Model (LLM) translations, and original Chinese texts across fifteen genres. Through analysis of three balanced comparable corpora, the research found that: (i) Compared to original Chinese texts, both human-translated and LLM-translated Chinese texts demonstrated significant syntactic simplification across all genres. (ii) Human translations exhibited a more pronounced tendency toward syntactic simplification than LLM translations across all genres. These findings not only validate the simplification hypothesis at the syntactic level but also highlight the different cognitive and processing mechanisms underlying human and LLM translation processes. The research indicates that human translators possess an active ability to optimize complex syntax that current LLMs lack, providing valuable reference for future development of LLMs and methods for LLM-assisted translation. Additionally, by adopting MDD and MHD as holistic measures of syntactic complexity, this study offers new perspectives for TU research and provides empirical insights into the linguistic nature of crowdsourcing translations from an English-to-Chinese perspective.

## Introduction

Benefiting from Baker’s pioneering research [1,2], corpus-based approaches to studying Translation Universals (TUs) gradually emerged. This new paradigm provided scientific basis for systematic research on translation features, marking a significant methodological shift in translation studies. Baker’s contribution to translation studies triggered a fundamental transformation: the research focus shifted from source text analysis to target system examination, and from the prescriptive concept of “equivalence” to the descriptive concept of “norms” [3]. The integration of corpus linguistics and translation studies enabled researchers to move beyond case studies toward empirical analysis of broader phenomena in translation. Chesterman [4,5] termed the comparative study of original texts and translated texts in the same language as TU research, aiming to reveal translators’ universal patterns of language use. Scholars have proposed various TUs, including explicitation, simplification, normalization, leveling out, interference, and the unique items hypothesis [6–8].

Over the past two decades, corpus-based TU research has proliferated, demonstrating academic value through methodological innovations. However, scholars have increasingly recognized methodological limitations in this field. As Kruger and van Rooy [9] note, language pairs and genres constitute two critical constraints in TU studies. Furthermore, despite the substantial impact of LLMs on contemporary translation practice, the textual and linguistic features of LLM-translated texts remain underexplored in computational linguistics and translation studies.

In light of this, this study aims to expand the depth and breadth of TU research by using MDD and MHD as indicators of syntactic complexity, comparing crowdsourcing human translations, LLM translations, and original Chinese texts across fifteen genres to evaluate simplification phenomena in English-to-Chinese translation. A comprehensive understanding and comparison of these translation patterns will enhance our knowledge of the distinct cognitive mechanisms underlying human and LLM translation processes, offering practical implications for effectively leveraging LLMs to improve translation efficiency.

Following this introduction, Section 2 reviews simplification research in human and LLM translation, and introduces the syntactic complexity metrics (MDD and MHD) and related studies. Section 3 details the corpus compilation methodology and data processing procedures. Section 4 presents the analytical results, while Section 5 provides theoretical interpretations through cognitive and related frameworks. Section 6 concludes with principal findings and suggests potential directions for future research.

## Literature review

### Simplification in human translation

According to Baker [6], simplification as one of the Translation Universals (TUs) can manifest in various ways, such as using shorter sentences, simpler vocabulary, or more direct syntactic structures. The fundamental assumption is that translators, whether consciously or unconsciously, typically attempt to reduce textual complexity to minimize comprehension barriers, making translated texts more accessible to the target audience. Since Baker proposed this concept, simplification has become one of the most frequently studied features in TU research.

Early TU research primarily focused on validating simplification at the lexical level. In a pioneering study, Laviosa [10] assessed lexical range through the ratio of high-frequency to low-frequency words and lexical density through the ratio of content words to function words, finding that translated texts exhibited lexical simplification characterized by narrower lexical ranges and lower lexical density compared to non-translated texts. Additionally, syntactic simplification has received attention: studies have explored longer mean sentence lengths [11], the use of non-standard collocations [12], and more frequent employment of modifiers [13]. Despite numerous attempts to establish metrics for measuring simplification and demonstrating the efficacy of computational linguistic techniques in studying translation features, consistent evidence remains elusive, and the simplification hypothesis continues to face skepticism. For instance, Xiao and Yue [14] observed that translated Chinese fiction exhibited significantly longer mean sentence lengths compared to native Chinese texts, suggesting that mean sentence length may not be an optimal indicator of simplification. Liu and Afzal [15] investigated differences between translated and native English using 13 metrics across five syntactic subconstructs from the L2 Syntactic Complexity Analyzer (L2SCA), yielding diverse and contradictory results across metrics. This inconsistency may stem from the excessive granularity of the metrics employed, highlighting the need for TU studies based on more holistic and macro-level indicators.

Regarding language-pair constraints, Xiao and Dai [16] noted that early TU research predominantly adopted a Eurocentric perspective, focusing on European language pairs. Investigating genetically distant language pairs, such as English-Chinese, may yield more compelling evidence for TUs. In recent years, growing awareness of these limitations has spurred increased TU studies on Chinese-English language pairs. For example, Liu and Afzaal [15] examined simplification in TU studies by comparing English translations from the Corpus of Chinese-English (COCE) with native English texts from the Freiburg-LOB Corpus of British English (F-LOB) using 13 syntactic complexity metrics. Niu and Jiang [17] explored simplification in specific text types by comparing Chinese translations with English source texts using lexical diversity as a metric. However, these studies primarily focus on Chinese-to-English translation processes, analyzing translated English texts. As Tan [18] observes, TU studies involving the reverse direction (English-to-Chinese translation) still requires further exploration.

Furthermore, early simplification research primarily concentrated on limited genres, neglecting the importance of fine-grained cross-genre corpora. For instance, numerous studies based on the Translational English Corpus (TEC) from University of Manchester primarily focused on literary genres, with insufficient attention to texts in other genres [15]. However, in corpus-based translation studies, genre is a key variable affecting the linguistic features of translated texts [9,19]. (Research by Delaere et al. [20] also indicates that both translation status and text type influence the composition of translated language. In recent years, some studies have begun to pay more attention to genre as a variable [15,17,21,22], but they typically adopt broad genre classification methods such as tripartite or quadripartite classifications rather than studying subtle differences between genres at higher granularity. For example, Niu & Jiang’s [17] simplification study analyzed only three genres (contemporary fiction, government documents, and academic abstracts); Liu and Afzaal’s [15] simplification study used a cross-genre corpus containing only four major genres—fiction, academic writing, general prose, and news—lacking distinction and observation between sub-genres within these major categories, thus unable to address issues such as differences in translation features between news reports and news commentaries. Therefore, simplification studies based on cross-genre corpora with higher granularity warrant further exploration.

### Simplification in LLM and crowdsourcing translation

Compared to the extensive research on simplification in human translation, studies on simplification in LLM-translated texts remain in their infancy. In recent years, ChatGPT has demonstrated exceptional capabilities across multiple domains, handling various conversational scenarios and content creation tasks, such as writing film reviews, composing poetry, and even correcting essays [23]. In the translation field, ChatGPT similarly shows tremendous potential. The model, pretrained on vast amounts of data and fine-tuned for specific translation tasks [24], can more effectively understand and process subtle differences between languages. Although ChatGPT was not specifically designed for translation, its translation potential can be fully leveraged through precise prompting. Several scholars, such as Gao et al. [25], Jiao et al. [26], and Lyu et al. [27], have reported potential improvements in ChatGPT’s translation quality compared to other commercial machine translation tools (such as Google Translate and DeepL). However, their primary focus has been on comparing translation performance between different LLMs based on metrics such as BLEU scores, and on how to prompt ChatGPT for better translations, rather than deeply analyzing the linguistic features of LLM-translated texts represented by ChatGPT. Currently, only a few studies have addressed the latter. Yim & Jin [28] examined how human translators, LLMs, and neural machine translation (NMT) handle long sentences in Korean-English translation of commercial reports, finding different strategies among the three entities in processing sentence boundaries for long sentences. Although this research is overly singular and narrow in terms of indicators and corpus genres, its findings suggest that LLM translation outputs differ significantly from traditional human translation and neural machine translation in linguistic features, warranting further in-depth and comprehensive consideration.

Since Jeff Howe first coined the term “crowdsourcing” in Wired magazine [29], the concept has rapidly permeated various industries. “Crowdsourcing” refers to outsourcing work traditionally completed by professionals to non-professional internet users [30]. Crowdsourcing translation is a concrete manifestation of the crowdsourcing concept in the translation industry in the era of the internet and big data. It is an entirely new translation model based on developments in information technology over the past 20 years, characterized by high efficiency and low cost, addressing to some extent the time-consuming nature of professional translator work [31]. This has led to its rapid and widespread application in translation practice: major platforms such as Google, Facebook, and Wikipedia have all called on users to participate in collaborative translation; popular online subtitle translation groups in recent years also represent another form of crowdsourcing translation. Some researchers even believe that crowdsourcing translation will become mainstream in the translation industry [32].

Since the integration of computational linguistics and descriptive translation studies, TUs in human professional translations have been extensively examined. However, as a computer-based translation technology distinct from human cognitive processes and a translation organizational form diverging from traditional translator roles [30], critical questions arise: Do LLM translations and crowdsourcing human translations align with TU hypotheses, such as simplification?

In summary, this study addresses the following research questions:

1. Do significant differences in syntactic complexity exist between translated Chinese and native Chinese texts, thereby supporting the simplification hypothesis? Are these findings consistent across genres?
2. Do both human-translated and LLM-translated Chinese exhibit simplification? If so, do they differ in the degree of simplification? Are these patterns consistent across genres?

## Methodology

### Corpus compilation

Delaere et al. [20] noted that simplification research typically employs comparable corpora composed of translated and original texts. To analyze differences among LLM-translated Chinese, human-translated Chinese, and original Chinese, and to examine the influence of genre on translation linguistic features, we selected three cross-genre balanced comparable corpora based on the paradigm. As shown in Table 1, these three corpora are the “Yiyan English-Chinese Parallel Corpus (Chinese monolingual section)” [33] (HT), the LLM translation corpus based on English monolingual section from Yiyan English-Chinese Parallel Corpus (O3), and the Texts Of Recent Chinese 2014 (ToRCH2014) (RC) [34]. They represent three text types: English-Chinese human translation, English-Chinese LLM translation, and original Chinese texts. These three corpora maintain balance across four major genres—fiction, academic writing, general prose, and news—and their 15 sub-genres, as shown in Table 2. The distribution proportions of genre samples across the three corpora are essentially consistent, constituting comparable corpora. Among them, the news genre (A-C) includes three sub-genres: news reports, editorials, and newspaper reviews; the general prose genre (D-H) comprises five sub-genres: religion, daily skills and recreational hobbies, popular readings, biographies and memoirs, and government or institutional documents and propaganda; the fiction genre (K-R) contains six sub-genres: general fiction, detective fiction, science fiction, adventure and suspense fiction, romantic fiction, and humorous fiction.

**Table 1:** Overview of the three corpora used in this study.

Abbreviation	Text Quantity	Corpus	Producer	Languages
HT	799	Yiyan corpus	Human translation	Chinese
O3	799	Yiyan corpus (translated)	ChatGPT O3-mini	Chinese
RC	657	ToRCH2014	Native speaker	Chinese

**Table 2:** Composition of the HT corpus.

Genre	Code	Sub-genre	Text Count	Chinese Character Count
News	A	News Reports	97	177513
	B	Editorials	48	106317
	C	Press Reviews	27	77073
General	D	Religion	31	62353
	E	Daily Skills and Entertainment	97	131160
	F	Popular Reading Materials	92	183636
	G	Biographies, Memoirs, etc.	121	293750
	H	Government/Institutional Documents	57	114614
Academic	J	Academic Works	82	308726
Fiction	K	General Fiction	57	188004
	L	Detective Fiction	13	43569
	M	Science Fiction	16	45893
	N	Adventure/Suspense Fiction	17	50027
	P	Romance Fiction	32	111814
	R	Humor	12	32739
Total			799	1927188

The Yiyan English-Chinese Parallel Corpus is a million-word balanced English-Chinese parallel corpus created according to the Brown Corpus model, containing 799 pairs of English-Chinese parallel texts totaling approximately 4 million words, with the vast majority of texts sourced from the crowdsourcing translation platform “Yeeyan (http://www.yeeyan.org).” We selected the Chinese monolingual section to form the HT corpus, representing crowdsourcing human translated Chinese.

The ToRCH2014 corpus contains approximately one million words and is one in the ToRCH (Texts Of Recent CHinese) corpus series. Its creators envisioned releasing a new version in the same pattern every few years to meet the need for examining the dynamic development of modern Chinese. All texts consist of original Chinese produced by native speakers of modern Chinese, serving as a comparison corpus for examining the linguistic features of translated Chinese.

For the O3 corpus construction, we extracted all English monolingual sections from the Yiyan English-Chinese Parallel Corpus and, using Python and the API provided by OpenAI, generated corresponding LLM-translated Chinese texts. To ensure the representativeness and cutting-edge nature of the selected LLM model, we followed the SOTA principle and selected O3-mini, the most advanced (as of March 8, 2025) Chain-of-Thought (CoT) LLM among OpenAI’s publicly released products.

### MDD and MHD as syntactic complexity metrics

As discussed in Section 2.1, overly granular metrics in TU studies may yield inconsistent or contradictory results, hindering a coherent understanding of TUs. To address this methodological challenge, MDD (Mean Dependency Distance) and MHD (Mean Hierarchical Distance) were adopted as holistic measures of syntactic complexity, quantifying differences across corpora. These metrics are grounded in dependency grammar, which posits three properties of syntactic dependencies: (1) binary, i.e., dependency relations between two syntactic elements; (2) asymmetric or directional, i.e., in two elements with a dependency relation, one element (the governor) dominates the other (the dependent), with the dependency direction pointing from the governor to the dependent; (3) marked, i.e., connecting two words with syntactic relations through arcs [35].

**Fig 1:**
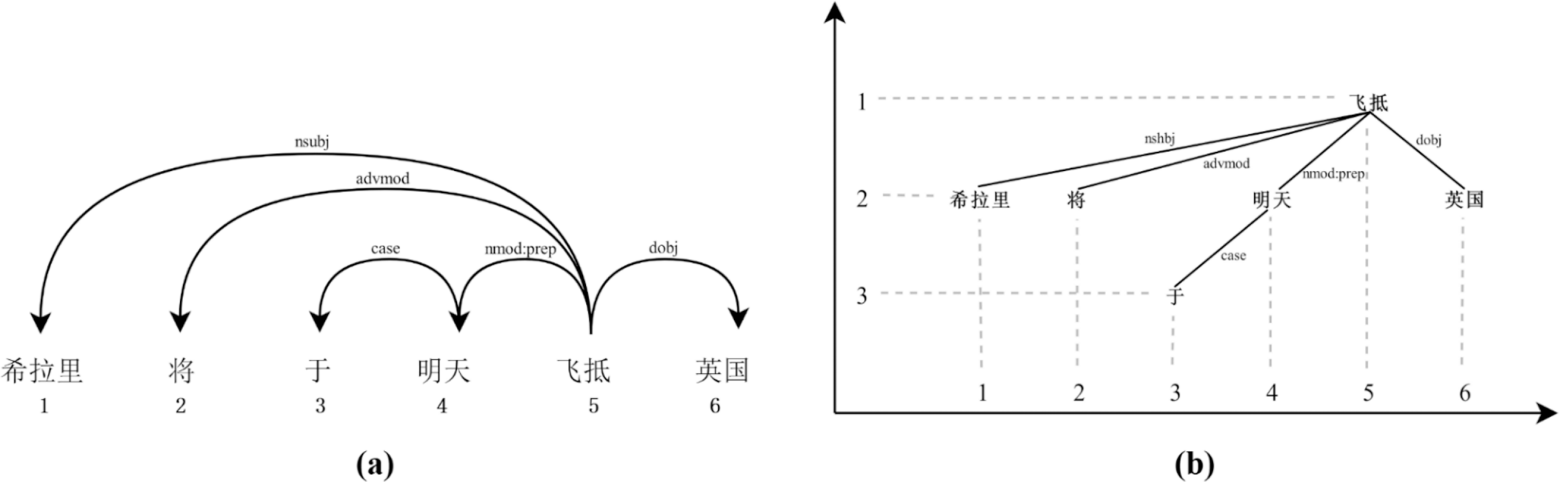
Two styles of dependency trees for the example sentence.

Taking the Chinese sentence “希拉里将于明天飞抵英国” (Hillary will fly to the UK tomorrow) as an example, Fig 1 shows two visualization methods for dependency relations: Fig 1(a) is the arrow diagram commonly used by computational linguists. In it, dependency relations are represented by directed arcs, with arrows pointing from the governor to the dependent; its advantage is clearly showing the types of dependency relations (above the arc) between words. Fig 1(b) is the syntactic tree commonly used by theoretical linguists. In it, dependency relations are represented by solid lines between words, with the governor in a relationship positioned at a higher level. The root word is at the highest level in the sentence. Dotted lines represent projection lines, projecting to both linear and hierarchical dimensions, with numbers representing the position of the word in these two dimensions. Thus, the advantage of it is a more intuitive reflection of the sentence hierarchy. Regardless of the method, both embody the core of dependency grammar as the connections between words (i.e., dependency relations), which is the basis for syntactic analysis and metrics calculation.

Building on this framework, Liu [36] and Jing&Liu [37] proposed MDD and MHD as indicators measuring the linear and hierarchical complexity of sentences, and confirmed their effectiveness across more than 20 natural languages. Subsequently, numerous studies have validated the effectiveness of MDD and MHD as key indicators for measuring memory load and syntactic complexity [38–40]. Jing and Liu [37] measured the linear and hierarchical structures of English and Czech based on MDD and MHD, and pointed out that MDD is a comprehension-oriented indicator, primarily measuring the difficulty of transforming from linear structure to hierarchical structure, while MHD is a production-oriented indicator, primarily measuring the difficulty of transforming from hierarchical structure to linear order.

At the word level, our method for calculating DD and HD follows Liu et al. [41] and Jing & Liu [37], expressed in formulas as follows:


DD = |Position(governor) − Position(dependent) (1)

That is, dependency distance equals the absolute value of the difference between the linear order of the governor and the dependent.


HD = Position(word) − Position(root) (2)

That is, hierarchical distance equals the difference between the level of a word and the level of the root word. As shown in Fig 1(b), the sentence root word is at level one, its dependents are at level two, and so on. Therefore, the hierarchical distance of a word can also be expressed as its level minus one, reflecting the difficulty of activating a word starting from the root word of a sentence.

At the sentence and discourse levels, the definitions of MDD and MHD are as follows:


$$MDD=\frac{1}{n}\sum \begin{array}{c} n\\ i=1 \end{array}DDi$$ (3)

Where n represents the total number of dependency relations in the sample (which can be a sentence, discourse, or the entire corpus), and DDi represents the dependency distance of the ith dependency relation. In other words, the MDD of a sample equals the arithmetic mean of the dependency distances of all dependency relations in the sample.


$$MHD=\frac{1}{n}\sum \begin{array}{c} n\\ i=1 \end{array}HDi$$ (4)

Where n represents the total number of dependency relations in the sample, and HDi represents the hierarchical distance of the ith dependency relation. In other words, the MHD of a sample equals the arithmetic mean of the hierarchical distances of all dependency relations in the sample.

Using the example sentence shown in Fig 1, the DD values of the six dependency relations in the sentence are 4, 3, 1, 1, 5, and 1, respectively. Therefore, according to formula (3), the sentence’s MDD = (4 + 3 + 1 + 1 + 5 + 1)/6 = 2.5. Additionally, excluding the root word, the HD values of the five tokens relative to the root word are 1, 1, 2, 1, and 1. Therefore, according to formula (4), the sentence’s MHD = (1 + 1 + 2 + 1 + 1)/5 = 1.2.

**Fig 2:**
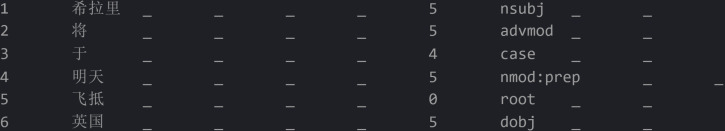
HanLP dependency parsing results for “希拉里将于明天飞抵英国” (CoNLL format).

To calculate the syntactic complexity of each text in the corpus, we first used the HanLP [42], a neural network-driven dependency grammar parser as a Python library, to parse the dependency structure of each sentence in the corpus. The parser identifies the governor, dependent, and dependency relation in each syntactic relation, and assigns a position code to each word in the sentence, ultimately returning a dependency treebank in CoNLL format (as shown in Fig 2). We then use Quansyn [43], a Python package oriented toward dependency grammar quantitative analysis, to calculate MDD and MHD sequentially at the sentence and text levels for the dependency treebank obtained in the previous step, ultimately obtaining the MDD and MHD values for each text in three corpora, and storing them in CSV files for subsequent statistical analysis.

### Statistical analysis

This study employed a 3 (text type: HT, O3, RC) × 15 (genre type: A-C, D-H, J, K-R) completely randomized two-factor experimental design, with the dependent variables being the measurement values of two syntactic complexity metrics. This method enabled comparative analysis in two dimensions: for research question 1, we conducted comparisons between translated Chinese and original Chinese (including HT vs. RC and O3 vs. RC); for research question 2, we conducted comparisons between human-translated Chinese and LLM-translated Chinese (HT vs. O3). To examine these three comparison groups both overall and across genres, we developed a comprehensive statistical analysis framework. The first part examines the three comparison groups overall to study general patterns of syntactic complexity; the second part examines the three comparison groups across fifteen sub-genres to understand how different genres influence the manifestation of translation features. This dual-perspective approach enables the identification and analysis of both macro-level patterns and genre-specific characteristics.

Prior to the main statistical analyses, preliminary data testing was conducted to determine appropriate statistical methods. For each indicator and comparison group, we first used the Shapiro-Wilk test to assess the normality of data distribution. Homogeneity of variance was then evaluated using Levene’s test. Based on these preliminary analyses, appropriate statistical methods were selected: Mann-Whitney U tests for non-normally distributed data; Student’s t-tests for normally distributed data with homogeneous variance; and Welch’s t-tests for normally distributed data with non-homogeneous variance. For the overall analysis of the three comparison groups, descriptive and inferential statistics were calculated. For normally distributed data, means and standard deviations were calculated; for non-normally distributed data, medians and interquartile ranges were reported. The genre-specific analysis followed the same statistical procedures but was conducted separately within each genre category. All statistical analyses were conducted at the 0.01 significance level.

To enhance the interpretability of statistical findings, we created violin plots using the R package ggplot2 [44] to visualize the distribution patterns of measurements between the three groups. Violin plots were chosen because they combine box plots with kernel density plots, comprehensively displaying data distribution, central tendency, and overall range. This visualization method provides detailed insights into syntactic complexity patterns across different text types.

## Results

### General analysis of the simplification hypothesis

#### General comparisons between translation and non-translation.

**Fig 3:**
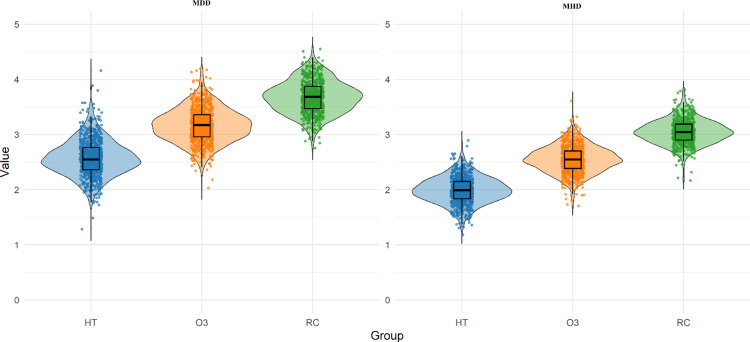
Violin plots of MDD&MHD distributions in HT, O3, and RC.

The results of MDD and MHD for texts in the three sub-corpora are shown in Fig 3 and Table 3. The three sub-corpora exhibit differences in MDD and MHD. Specifically, RC demonstrates the highest values in both MDD and MHD measurements, followed by O3, and finally HT.

**Table 3:** Descriptive statistics and statistical comparison of MDD and MHD between translated and non-translated texts.

Group	Metrics	Type			Test	p
		HT	O3	RC		
HT vs O3	MDD	2.55 [0.4]	3.17 [0.4]	–	Mann-Whitney U Test	<0.01
	MHD	1.99 (0.24)	2.55 (0.24)	–	Student’s t-test	<0.01
HT vs RC	MDD	2.55 [0.4]	–	3.68 [0.4]	Mann-Whitney U Test	<0.01
	MHD	1.99 [0.31]	–	3.04 [0.29]	Mann-Whitney U Test	<0.01
O3 vs RC	MDD	–	3.17 (0.31)	3.68 (0.3)	Student’s t-test	<0.01
	MHD	–	2.55 [0.32]	3.04 [0.29]	Mann-Whitney U Test	<0.01

Table 3 provides a summary of descriptive statistics and differential analysis results(note: values in Tables 3 and 4 are reported as mean (standard deviation) or median [interquartile range]). Statistical analysis confirms that the differences between both types of translated texts and non-translated texts are significant (p < 0.01 for both HT vs. RC and O3 vs. RC comparisons). The lower MDD and MHD values in HT and O3 strongly support the simplification hypothesis, indicating that both human translator and LLM tend to produce sentences with lower MDD and MHD during the translation process, thereby making the translation output simpler compared to non-translated text.

**Table 4:** MDD&MHD calculation results of cross-genre analysis.

Genre	Metrics	Type		
		HT	O3	RC
A	MDD	2.6 [0.38]	3.19 [0.3]	3.72 [0.32]
	MHD	2.14 (0.23)	2.71 (0.23)	3.19 (0.23)
B	MDD	2.57 [0.33]	3.24 (0.24)	3.76 (0.26)
	MHD	2.14 (0.18)	2.7 (0.18)	3.11 (0.17)
C	MDD	2.82 (0.21)	3.48 (0.19)	3.86 (0.25)
	MHD	2.2 (0.14)	2.74 (0.18)	3.17 (0.18)
D	MDD	2.59 [0.28]	3.22 (0.2)	3.65 (0.29)
	MHD	2.02 (0.24)	2.57 (0.24)	3.04 (0.21)
E	MDD	2.4 (0.29)	2.95 (0.29)	3.69 (0.28)
	MHD	1.85 [0.27]	2.33 [0.26]	2.99 [0.19]
F	MDD	2.58 (0.3)	3.17 (0.26)	3.56 (0.24)
	MHD	1.94 (0.2)	2.5 (0.2)	2.93 (0.17)
G	MDD	2.57 [0.34]	3.17 (0.24)	3.56 (0.2)
	MHD	1.98 (0.16)	2.54 (0.18)	2.95 (0.13)
H	MDD	2.64 (0.37)	3.22 (0.34)	3.96 (0.19)
	MHD	2.12 [0.29]	2.67 [0.27]	3.23 [0.21]
J	MDD	2.87 [0.35]	3.44 (0.3)	3.94 (0.22)
	MHD	2.13 (0.18)	2.67 (0.18)	3.18 (0.13)
K	MDD	2.39 [0.44]	3.02 (0.28)	3.46 (0.22)
	MHD	1.84 (0.17)	2.41 (0.18)	2.89 (0.13)
L	MDD	2.39 [0.48]	2.97 (0.38)	3.43 (0.19)
	MHD	1.78 (0.26)	2.37 (0.28)	2.88 (0.11)
M	MDD	2.38 (0.26)	2.93 (0.27)	3.36 (0.25)
	MHD	1.81 (0.21)	2.35 (0.19)	2.81 (0.16)
N	MDD	2.43 (0.33)	3.04 (0.33)	3.54 (0.23)
	MHD	1.87 (0.21)	2.44 (0.21)	2.95 (0.16)
P	MDD	2.39 (0.26)	3.1 (0.31)	3.41 (0.23)
	MHD	1.8 (0.2)	2.41 (0.22)	2.84 (0.16)
R	MDD	2.31 (0.36)	2.94 (0.4)	3.17 (0.28)
	MHD	1.81 [0.14]	2.35 (0.21)	2.63 (0.33)

#### General comparisons between human translation and LLM translation.

The comparison between HT and O3 reveals significant differences in their syntactic complexity. According to Table 3, O3 displays higher levels of syntactic complexity in both MDD and MHD (MDD = 3.17 [0.4], MHD = 2.55 (0.24)), compared to the lower values in HT (MDD = 2.55 [0.4], MHD = 1.99 (0.24)).

When comparing both translation types to RC (MDD = 3.68 (0.3), MHD = 3.04 [0.29]), it can be observed that both HT and O3 exhibit simplification features, but to different degrees. Human translation shows a more pronounced simplification pattern, with MDD reduced by approximately 31% and MHD reduced by approximately 35% compared to RC. Although LLM translation also demonstrates simplification, the reduction is smaller, with MDD approximately 14% lower and MHD approximately 16% lower than RC.

These findings suggest that while both human translation and LLM translation support the simplification hypothesis, the degree of simplification differs between the two translation types. Compared to LLMs, human translators tend to simplify syntactic structures more extensively.

### Cross-genre analysis of the simplification hypothesis

#### Cross-genre comparisons between translation and non-translation.

**Fig 4:**
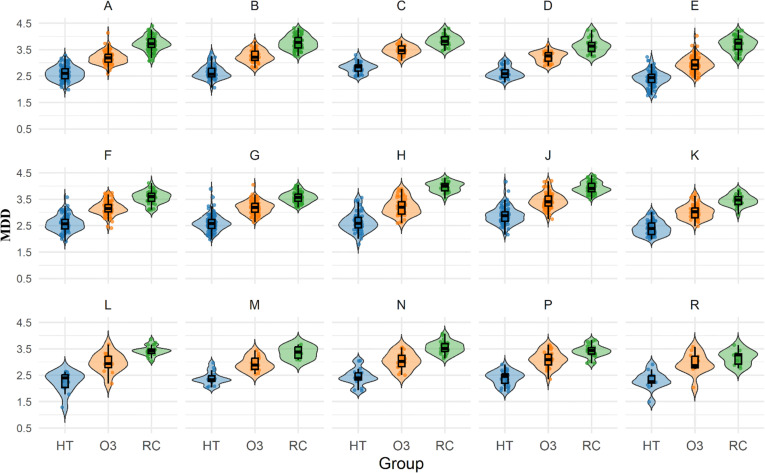
Violin plots of MDD distributions across 15 genres in HT, O3, and RC.

**Fig 5:**
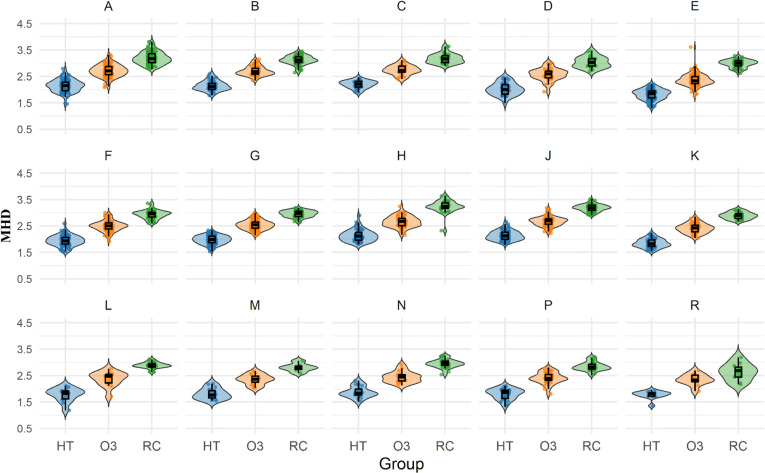
Violin plots of MHD distributions across 15 genres in HT, O3, and RC.

To investigate whether the simplification effect remains consistent across different genres, we conducted genre-specific analyses. Figs 4,5, and Table 4 show the calculated results of MDD and MHD for HT, O3, and RC across 15 different genres, while Table 5 presents a summary of the descriptive statistics and differential analysis results from the genre-specific analyses.

**Table 5:** Test and p value results of cross-genre analysis.

Genre	Metrics	Comparison Group					
		HT vs O3		HT vs RC		O3 vs RC	
		Test	p	Test	p	Test	p
A	MDD	Mann-Whitney U Test	<0.01	Student’s t-test	<0.01	Mann-Whitney U Test	<0.01
	MHD	Student’s t-test	<0.01	Student’s t-test	<0.01	Student’s t-test	<0.01
B	MDD	Mann-Whitney U Test	<0.01	Mann-Whitney U Test	<0.01	Student’s t-test	<0.01
	MHD	Student’s t-test	<0.01	Student’s t-test	<0.01	Student’s t-test	<0.01
C	MDD	Student’s t-test	<0.01	Student’s t-test	<0.01	Student’s t-test	<0.01
	MHD	Student’s t-test	<0.01	Student’s t-test	<0.01	Student’s t-test	<0.01
D	MDD	Mann-Whitney U Test	<0.01	Mann-Whitney U Test	<0.01	Student’s t-test	<0.01
	MHD	Student’s t-test	<0.01	Student’s t-test	<0.01	Student’s t-test	<0.01
E	MDD	Student’s t-test	<0.01	Student’s t-test	<0.01	Student’s t-test	<0.01
	MHD	Mann-Whitney U Test	<0.01	Welch’s t-test	<0.01	Mann-Whitney U Test	<0.01
F	MDD	Student’s t-test	<0.01	Student’s t-test	<0.01	Student’s t-test	<0.01
	MHD	Student’s t-test	<0.01	Student’s t-test	<0.01	Student’s t-test	<0.01
G	MDD	Mann-Whitney U Test	<0.01	Mann-Whitney U Test	<0.01	Student’s t-test	<0.01
	MHD	Student’s t-test	<0.01	Welch’s t-test	<0.01	Welch’s t-test	<0.01
H	MDD	Student’s t-test	<0.01	Welch’s t-test	<0.01	Welch’s t-test	<0.01
	MHD	Mann-Whitney U Test	<0.01	Mann-Whitney U Test	<0.01	Mann-Whitney U Test	<0.01
J	MDD	Mann-Whitney U Test	<0.01	Mann-Whitney U Test	<0.01	Welch’s t-test	<0.01
	MHD	Student’s t-test	<0.01	Welch’s t-test	<0.01	Welch’s t-test	<0.01
K	MDD	Mann-Whitney U Test	<0.01	Mann-Whitney U Test	<0.01	Student’s t-test	<0.01
	MHD	Student’s t-test	<0.01	Student’s t-test	<0.01	Student’s t-test	<0.01
L	MDD	Mann-Whitney U Test	<0.01	Mann-Whitney U Test	<0.01	Welch’s t-test	<0.01
	MHD	Student’s t-test	<0.01	Welch’s t-test	<0.01	Welch’s t-test	<0.01
M	MDD	Student’s t-test	<0.01	Student’s t-test	<0.01	Student’s t-test	<0.01
	MHD	Student’s t-test	<0.01	Student’s t-test	<0.01	Student’s t-test	<0.01
N	MDD	Student’s t-test	<0.01	Student’s t-test	<0.01	Student’s t-test	<0.01
	MHD	Student’s t-test	<0.01	Student’s t-test	<0.01	Student’s t-test	<0.01
P	MDD	Student’s t-test	<0.01	Student’s t-test	<0.01	Student’s t-test	<0.01
	MHD	Student’s t-test	<0.01	Student’s t-test	<0.01	Student’s t-test	<0.01
R	MDD	Student’s t-test	<0.01	Student’s t-test	<0.01	Student’s t-test	0.15
	MHD	Mann-Whitney U Test	<0.01	Mann-Whitney U Test	<0.01	Student’s t-test	0.02

Except for Genre R in the O3-RC comparison (non-significant difference), all three comparison groups (HT vs. RC, O3 vs. RC, HT vs. O3) exhibited consistent simplification patterns across genres (p < 0.01 for 15 genres and both metrics), providing strong support for the simplification hypothesis in different genres. Specifically, in almost every genre (A through R), both HT and O3 consistently display lower MDD and MHD values compared to RC (see Table 4), and statistical analysis confirms (see Table 5) that the differences within both comparison groups are significant (p < 0.01 for both HT vs. RC and O3 vs. RC comparisons across fifteen genres). This indicates that the tendency toward syntactic simplification in translated texts is not limited to specific genres but is a universal phenomenon across various genres.

Additionally, we observed that although both HT and O3 consistently exhibit simplification features compared to RC across all genres, the degree of simplification varies. Human translation demonstrates the most pronounced simplification across all genres. For example, in religious texts (genre D), HT (MDD = 2.59 [0.28], MHD = 2.02 (0.24)) shows approximately 30% lower MDD and 34% lower MHD compared to RC (MDD = 3.65 (0.29), MHD = 3.04 (0.21)). While O3 also exhibits simplification compared to RC, the degree is less pronounced than HT. Using the same religious texts (genre D) as an example, O3 (MDD = 3.22 (0.2), MHD = 2.57 (0.24)) shows approximately 12% lower MDD and 15% lower MHD compared to RC.

#### Cross-genre comparisons between human translation and LLM translation.

The genre-specific analysis of human translation and machine translation reveals consistent patterns across the 15 examined genres, as detailed in Table 5. In all genres, MT consistently displays higher MDD values than HT, indicating higher levels of syntactic complexity in machine-translated texts across diverse text types.

When comparing both translation types to the NT within each genre, we observe that both HT and MT consistently exhibit simplification features across all text types. However, the degree of simplification varies. Human translation demonstrates the most pronounced simplification across all genres. For instance, in religious texts (Genre D), HT (M = 1.78, IQR = 0.48) shows a 43.7% reduction in MDD compared to NT (M = 3.16, IQR = 0.34).

Machine Translation, while still exhibiting simplification compared to NT, shows less pronounced patterns of simplification. Using the same example of religious texts (Genre D), MT (M = 2.59, IQR = 0.58) shows an 18% reduction in MDD compared to NT. This consistent pattern across genres suggests that the differences in syntactic complexity between HT and MT are not genre-specific in crowd-sourced Yiyan translation.

## Discussion

### Overall simplification and cross-genre simplification

The overall syntactic simplification observed in this study may be influenced by “source language interference,” whereby “phenomena pertaining to the make-up of the source text tend to be transferred to the target text” [45]. Compared to English, Chinese lacks inflectional morphology and thus relies more heavily on syntactic mechanisms to express grammatical relations such as tense, number, and case. This characteristic of compensating for morphological simplicity through syntactic complexity typically makes Chinese texts more syntactically complex than English texts [46]. Since most of the linguistic material in our corpus was translated from English, during the translation process, the more concise English structures would transfer to translated Chinese through the “source language interference” effect, resulting in structural compression and more simplified syntactic structures in the latter.

The Hypothesis of Gravitational Pull [47,48] based on bilingual theory and cognitive linguistics further elucidates this process. This hypothesis posits that typical or salient linguistic forms in the target language influence translators’ decisions, producing a “ magnetism effect”; conversely, the source text generates a counterforce resisting this attraction, leading to interference, termed the “gravitational pull effect.” A third effect, the “connectivity effect,” stems from high-frequency co-occurrences of translation equivalents in source and target languages. The interaction of these three forces shapes translation language. This study indicates that in English-Chinese translation, the gravitational effect is more prominent among the three forces, causing translated texts to more frequently exhibit simpler syntactic structures from English source texts, ultimately making translated Chinese more concise compared to original Chinese texts. This aligns with Liu & Afzaal’s [15] findings that the gravitational effect has a greater influence than the other two effects in forming translation language.

Furthermore, this study’s findings indicate that translated Chinese exhibits smaller MDD and MHD values than original Chinese across all genres, thus confirming the simplification hypothesis across all genres studied. This result differs from some previous studies. For instance, Liu et al. [22], using syntactic entropy as a complexity indicator, observed complexification in human translation of academic genres. This difference can be explained based on the different nature of the corpora used and Pym’s hypothesis of risk avoidance [49]. *The Yiyan English-Chinese Parallel Corpus* used for human translation in this study consists of crowdsourcing translations from *Yeeyan (yeeyan.org)*, most of which were completed by non-professional translators for online publication. In contrast, *Zhe Jiang University Corpus of Translational Chinese* (ZCTC) used by Liu et al. [22] primarily comprises professionally translated works intended for formal publication. Academic texts pose unique challenges to translators due to their specialized readership and inherent complex discourse. According to Pym’s framework, professional translators working on academic works intended for formal publication tend to adhere more strictly to source text styles to minimize the risk of misunderstanding [50], which undoubtedly enhances the “source language interference” effect from complex English structures, thereby increasing the likelihood of producing more complex sentences. In contrast, crowdsourcing translation represents a collaborative translation model by non-professional freelance translators for online distribution, where translators face relatively less pressure in risk management during the translation process, thus tending to follow innate tendencies in human cognition such as the principle of least effort.

### Differences between LLM translation and human translation

The phenomenon observed in this study, where human translations exhibit a higher degree of simplification than LLM translations, suggests that human translators often demonstrate better active optimization capabilities than LLMs when facing complex syntactic structures. Zhang et al. [51] evaluated the performance of humans and LLMs in translating literary texts in English-German, German-English, English-Chinese, and German-Chinese language pairs, pointing out that LLM outputs tend toward literal translation, lacking diversity and nuance, with this gap being particularly evident in texts involving long-distance contexts, such as literary works. A possible reason is that LLMs, constrained by their self-attention algorithm when executing translation tasks, excessively pursue local translation accuracy without incorporating factors such as syntactic conciseness and reader acceptance based on context and pragmatics. In contrast, human translators, through strategies such as analyzing context, keywords, and themes, can more effectively address highly complex texts, demonstrating the advantage of humans over LLMs in active cognitive engagement [52]. Li and Hu [21] also indicate that by comparing human and machine translation, human translators can flexibly adjust translation strategies according to different contexts and text types while consciously optimizing language structures during the translation process. This active optimization capability underlies human translations’ greater simplification.

Additionally, the characteristic of computer algorithms like LLMs having no cognitive burden may be an important reason for their lower degree of simplification. In the context of language universals, Dependency Distance Minimization (DDM) represents a universal characteristic of human languages. Liu’s [36] research based on multilingual dependency treebanks found that the difficulty of language processing positively correlates with dependency distance: the longer the dependency distance, the greater the processing difficulty, revealing the existence of the DDM phenomenon. Subsequently, DDM has been verified in multiple languages and scenarios, including synchronically [36,53,54] and diachronically [39], and has been proposed as a universal language law [38]. This indicates that human language processing systems prefer shorter dependency distances, and this preference becomes more pronounced in high cognitive load situations such as translation—rapid language switching and bilingual activation during the translation process subject translators to additional cognitive load [55], making them more inclined to adopt simpler syntactic structures to reduce the difficulty of processing source language and outputting target language, ensuring efficient information transmission. This selection mechanism also aligns with Zipf’s [56] principle of least effort—humans naturally choose ways with lower cognitive costs when using language. In contrast, LLM, as computer algorithms, unburdened by cognitive constraints when performing translation tasks and processing complex sentences. This “burden-free” characteristic means that LLM lack intrinsic motivation to optimize complex structures when generating translations.

The following examples from our corpora demonstrate how human translators flexibly adapt syntax to reduce complexity, contrasting with LLMs’ structural conservatism.

Example 1 (Genre A):

Original English: As a Senator, Hillary Clinton authored a Foreign Affairs article in which she stated the U.S.--China relationship was the most important relationship in Asia, rekindling Japanese angst from the slight suffered when President Bill Clinton traveled to China but skipped Japan.

HT: 作为参议员，希拉里克林顿在所写的“国际事务”的文章中说到美中关系是亚洲地区最为重要的关系。在比尔克林顿总统当政期间他就访问北京而跳过了日本，从而使得日本小小地伤心了一把，希拉里此文不禁重新点燃了他们的担心。(MDD: 2.9434, MHD: 2.2264)

(Literal Translation: As a senator, Hillary Clinton stated in an article she authored for ‘Foreign Affairs’ that the U.S.-China relationship was the most important in Asia. During President Bill Clinton’s tenure, he visited Beijing but skipped Japan, thereby making Japan feel slightly hurt. Hillary’s article inadvertently reignited their concerns.)

O3: 早在担任参议员时，希拉里·克林顿就在一篇关于外交事务的文章中指出，美中关系是亚洲最重要的关系，这无疑重新激起了日本对克林顿父亲比尔·克林顿总统当年访华却忽略日本时受到的小小冒犯的不满。(MDD: 4.1224, MHD: 3.6122)

(Literal Translation: Early in her tenure as a senator, Hillary Clinton pointed out in an article on foreign affairs that the U.S.-China relationship was the most important in Asia, which undoubtedly reignited Japan’s resentment over the minor slight suffered when President Bill Clinton, her father, visited China but ignored Japan.)

In this example from News genre, the English source text features a complex sentence with embedded clauses and coordinate structures. The human translator identifies two primary information units (Hillary’s article and Japan’s reaction) and strategically divides the original compound sentence into two independent short clauses. By reorganizing the information sequence (postposing “希拉里此文不禁重新点燃了他们的担心” [“Hillary’s article inadvertently reignited their concerns “]) and inserting logical connectives (e.g., “从而” [“thereby”]), the translator effectively reduces dependency distances between modifiers and their head verbs, achieving a 29% decrease in MDD and 38% reduction in MHD compared to the LLM output. Furthermore, the human translator employs localized expressions such as “小小地伤心了一把” (“feel slightly hurt “) to enhance readability. In contrast, the LLM translation mechanically preserves the original syntactic framework, attempting to compress all information into a single complex sentence. This results in a typical nested syntactic structure (“重新激起了日本对...的不满” [“which undoubtedly reignited... but ignored Japan “]) with elongated dependency chains, significantly increasing cognitive processing load.

Example 2 (Genre K):

Original English: Dad comes up from the basement in his gimpy comic trot, concerned, takes a bullet in the chest, drops to his knees, takes one in the head, and that’s that.

HT: 我爸一瘸一拐地从地下室里小跑上来，结果胸口挨了一枪，跪倒在地。接着头上又中了一枪，就这样完了。(MDD: 2.4138, MHD: 1.6552)

(Literal Translation: *My dad limped up from the basement in a comically hurried trot; as a result, he took a bullet in the chest and dropped to his knees. Next, he took another bullet in the head, and that was that.*)

O3: *爸爸从地下室拄着拐杖踉跄上来，表情既滑稽又忧虑，却也中了胸部一枪，随即跪倒在地，再中了一枪打在头上，就这样，完了。*(MDD: 3.4242, MHD: 2.1212)

(Literal Translation: *Dad, leaning on a crutch, staggered up from the basement with an expression both comical and worried, yet took a bullet in the chest, immediately knelt down, then took another bullet to the head, and thus, it was over.*)

In this example from fiction, the human translator demonstrates active optimization of discourse structure and precise control of narrative rhythm once more. The HT translation uses four-character phrases (“一瘸一拐[limped up]”), verb sequences (“挨[took]、跪[dropped to his knees]、中[took]”), and other Chinese-specific expressions to transform the original parallel verb structure into a chronologically clear flowing sentence, resulting in a dependency distance (MDD = 2.41) that is 29.5% lower than the LLM translation (3.42), while meeting the fiction genre’s requirements for action fluidity and reader immersion. Particularly noteworthy is how the human translator creatively renders the original description of the father’s movement, “gimpy comic trot,” as “一瘸一拐地从地下室里小跑上来(limped up from the basement in a comically hurried trot)”, both preserving the dark humor of the original and constructing “temporal iconicity” [57] consistent with Chinese cognitive habits through spatial order (“从地下室[from the basement]→上来[up]”) and linear arrangement of the action chain (“跑[run]→挨枪[take bullet]→跪倒[kneel]”). In contrast, the LLM translation lacks active adaptation to context, exhibiting the phenomenon of “excessive pursuit of local translation accuracy” mentioned above through its rigid adherence to source-text syntax, as seen in the front-loaded modifiers “拄着拐杖踉跄上来，表情既滑稽又忧虑(leaning on a crutch, staggered up from the basement)”. This mechanical alignment extends dependency distances between “上来(up)” and the main verb “中枪(took a bullet)”, disrupting action continuity and producing stylistically awkward outputs that compromise narrative naturalness.

These examples reveal fundamental distinctions between human and LLM translation: Human translation constitutes creative reconstruction guided by cognitive economy principles, enabling strategic adjustments based on genre characteristics—prioritizing conceptual clarity in information-dense texts while emphasizing event-chain fluency in narratives. LLM translation, conversely, operates through statistical surface-structure mapping lacking metalinguistic genre awareness. When processing high-density information, human translators activate cognitive compensation mechanisms: converting long-distance dependencies into local dependencies via clause segmentation (as in Example 1), or reorganizing information units through exploitation of Chinese paratactic features (as in Example 2). This dynamic adaptability stems from human translators’ profound understanding of cross-linguistic cognitive framework disparities [47]. LLM, constrained by their self-attention-based processing architecture, systematically prioritize source-structure preservation. When confronting English-Chinese syntactic conflicts, their structural conservatism manifests in preference for extended modification over structural reorganization, thereby passively increasing syntactic complexity.

## Conclusion

This study employs MDD and MHD as syntactic complexity metrics to investigate syntactic simplification in translation. By comparing MDD and MHD values across crowdsourcing human-translated Chinese, LLM-translated Chinese and native Chinese texts in 15 genres, we demonstrate that translated Chinese exhibits significantly lower syntactic complexity than original Chinese in nearly all genres, thus further strengthening the evidence for simplification as a Translation Universal. Furthermore, crowdsourcing human translations consistently show greater simplification than LLM translations across all genres, indicating that human translator actively optimize syntactic structures during translation, while LLM remain constrained by limitations in handling syntactically complex texts. Our analysis of LLM-translated linguistic features offers insights for optimizing LLM development and refining LLM-assisted translation methodologies.

However, this study has limitations that warrant attention. First, the LLM analysis is based solely on OpenAI’s O3-mini model. While this model represents state-of-the-art LLM technology, variations in architectural design, training data composition, and decoding strategies across different LLMs (e.g., GPT-4, Gemini, Claude) may lead to divergent syntactic patterns. Consequently, the identified simplification phenomena should be interpreted as architecture-specific manifestations rather than universal LLM characteristics. Future research imperatively requires comprehensive comparative analyses across heterogeneous LLM to establish the cross-model validity of simplification. Second, it is important to note that this study focuses exclusively on English-to-Chinese translation. Future studies should expand to other language pairs to advance our understanding of simplification across linguistic and contextual variations. Additionally, while MDD and MHD demonstrate high validity as syntactic complexity metrics, future research could integrate complementary measures (e.g., information entropy) to explore simplification phenomena in human and LLM translations across multiple dimensions.

## Supporting information

S1 File Self-built yiyan O3-mini corpus. (ZIP)
